# Supplementary material for: SpeciesPrimer: a bioinformatics pipeline dedicated to the design of qPCR primers for the quantification of bacterial species
Source: PeerJ. 2020 Feb 18;8:e8544. doi: 10.7717/peerj.8544 (PMC7034379; doi:10.7717/peerj.8544)
Supplement: Dataset S5 — Summarized alignments of web-based BLAST search against the RefSeq Genomes database (limited to Bacteria, taxid:2) with the predicted PCR product of the primer pairs as query. The first line in the alignment represent the forward primer sequence (left) and the reverse-complement sequence of the reverse primer (right). The second line represents the predicted PCR product. Only one sequence per BLAST hit for a certain species is shown if the sequences were identical, the number of these redundant hits is given in the last row of the results table. [file peerj-08-8544-s005.pdf]

Summarized results of predicted amplicon BLAST search against the RefSeq Genomes database (limited to Bacteria, taxid:2).

Summarized alignments of web-based BLAST search with the predicted PCR product of the primer pairs as query. The first line in the alignment represent the forward primer sequence (left) and the reverse-complement sequence of the reverse primer (right). The second line represents the predicted PCR product. Only one sequence per BLAST hit for a certain species is shown if the sequences were identical, the number of these redundant hits is given in the last row of the results table.

Ec_faeca_acuI_1_P0

```
Ecfaeca_acuI_P0    1 TCATTTCAAGCATTTACGTTAAGAGA---------------------TCAACTAGAAACC
query_acuI_1       1 TCATTTCAAGCATTTACGTTAAGAGAACAAGNTACATCTGTTATTGGTCAACTAGAAACC
BLASTHIT_1         1 TCATTTCAAGCATTTACGTTAAGAGAACAAGATACATCTGTTATTGGTCAACTAGAAACC
BLASTHIT_2         1 TCATTTCAAGCATTTACGTTAAGAGAACAAGATACATCTGTTATTGGTCAACTAGAAACC
BLASTHIT_3         1 TCATTTCAAGCATTTACGTTAAGAGAACAAGGTACATCTGTTATTGGTCAACTAGAAACC
BLASTHIT_4         1 TCATTTCAAGCATTTACGTTAAGAGAACAAGATACATCTGTCATTGGTCAACTAGAAACC
BLASTHIT_5         1 TCATTTCAAGCATTTACGTTAAGAGAACAAGACACATCTGTTATTGGTCAACTAGAAACC
BLASTHIT_6         1 TCATTTCAAGCATTTACGTTAAGAGAACA-------------------------------
BLASTHIT_7         1 -----------------------------------------TATTGGTCAAGTTGAGACC
BLASTHIT_8         1 ------------------------GAAGAAGATTCACCTGTTATTGGTCAAGTAGACGCC
BLASTHIT_9         1 ------------------------GAAGAAGATTCACCTGTTATTGGTCAAGTAGACGCC
BLASTHIT_10        1 --------------------------------------------TGGTCAACGAGAAACC
BLASTHIT_11        1 --------------------------------TACATCTGTTATTGGTCGACCAGAAA--
BLASTHIT_12        1 -------------------------------------------TTGGTCAACTAGCAACT
BLASTHIT_13        1 --------------------------ACAAGGAACATCTGTTATTGGTCAA---------
BLASTHIT_14        1 --------------------AAAAGAACAGGTTACTTTTGTTATTGGTCATCCAGTAACC
BLASTHIT_15        1 --ATTCCAAGCATTTACATTTAGAAGAGAACAAGGTAC----------------------
BLASTHIT_16        1 ----------------------------------------TTCTTGATCAACTTGACACC
BLASTHIT_17        1 ----------------------------------CATCTGTTATTGGTCAACTA------
BLASTHIT_18        1 ------CAAGCATTTACGTTAAGAGA----------------------------------
BLASTHIT_19        1 -----------------------------------------------TCAACTTGAAACG
BLASTHIT_20        1 --------------TACGTTAAGAGAACAAGATACA------------------------
...                1 ------------------------------------------------------------
...                1 ------------------------------------------------------------
N=96               1 ------------------------------------------------------------

Ecfaeca_acuI_P0   40 ATTACTTTAGACG
query_acuI_1      61 ATTACTTTAGACG
BLASTHIT_1        61 ATTACTTTAGACG
BLASTHIT_2        61 ATTACTTTAGACG
BLASTHIT_3        61 ATTACTTTAGACG
BLASTHIT_4        61 ATTACTTTAGACG
BLASTHIT_5        61 ATTACTTTAGACG
BLASTHIT_6           -------------
BLASTHIT_7        20 ATTACTTTAGA--
BLASTHIT_8        37 ATT----------
BLASTHIT_9        37 ATT----------
BLASTHIT_10       17 ATTACTTT-----
BLASTHIT_11          -------------
BLASTHIT_12       18 ATTATTTTAGA--
BLASTHIT_13          -------------
BLASTHIT_14       41 -------------
BLASTHIT_15          -------------
BLASTHIT_16       21 ATTACTTTAG---
BLASTHIT_17          -------------
BLASTHIT_18          -------------
BLASTHIT_19       14 ATTACTTTAGAC-
BLASTHIT_20          -------------
...                  -------------
...                  -------------
N=96                 -------------
```

BLAST HIT	Species	Query coverage [%]	Identity [%]	Accession	redundant hits
1	Enterococcus faecalis	100	99	NC_004668.1	690 x
2	Enterococcus sp.	100	99	NZ_JWBQ01000067.1	3 x
3	Enterococcus faecalis	100	99	NZ_JUXC01000095.1	-
4	Enterococcus faecalis	100	97	NZ_PHLF01000001.1	-
5	Enterococcus faecalis	100	97	NZ_KZ845811.1	-
6	Staphylococcus aureus	40	100	NZ_LJOC01000115.1	1 x
7	Anaerorhabdus furcosa	41	90	NZ_FUWY01000002.1	-
8	Oceanihabitans sediminis	53	82	NZ_QPIG01000005.1	1 x
9	Flavobacteriaceae bacterium	53	82	NZ_MPGM01000017.1	-
10	Nitrosotalea sp.	33	96	NZ_FRFC01000001.1	-
11	Collimonas fungivorans	36	92	NZ_CP013232.1	-
12	Nostoc piscinale	38	89	NZ_CP012036.1	-
13	Ewingella americana	34	92	NZ_JMPJ01000065.1	-
14	Neisseriaceae bacterium	55	80	NZ_CP024847.1	-
15	Clostridium kluyveri	46	80	NZ_CP018335.1	-
16	Planktothrix agardhii	41	87	NZ_CM002803.1	-
17	Hungateiclostridium cellulolyticum	27	100	NZ_JH556653.1	-
18	Pseudoalteromonas atlantica	27	100	NC_008228.1	-
19	Bacillus simplex	34	92	NZ_NQLS01000166.1	2 x
20	Olleya namhaensis	30	95	NZ_FORM01000001.1	-

* anomalous sequences excluded from alignment
gi|1423449487|ref|NZ_UEMU01000447.1|Escherichia coli strain KCRI-77E isolate RDK43_77E
gi|1005608163|ref|NZ_FFHQ01000001.1|Listeria monocytogenes strain 2842STDY5753961
gi|522836524|ref|NZ_KE352150.1|Enterococcus faecalis strain SB2C-2 Scaffold11
gi|1120478950|ref|NZ_FQTZ01000002.1|Enterococcus faecium isolate Hp_76-17_S13_

Ec_faeca_g3060_1_P0

```
Ecfaeca_g3060_P     1 CCCTCTTTAACATTAATTGGACTTGC----------------------AGCCAAGCCTAT
query_g3060_1       1 CCCTCTTTAACATTAATTGGACTTGCNCAGCAATTATTTATTAGTAAAAGCCAAGCCTAT
BLASTHIT_1          1 CCCTCTTTAACATTAATTGGACTTGCCCAGCAATTATTTATTAGTAAAAGCCAAGCCTAT
BLASTHIT_2          1 CCCTCTTTAACATTAATTGGACTTGCCCAGCAATTATTTATTAGTAAAAGCCAAGCCTAT
BLASTHIT_3          1 CCCTCTTTAACATTAATTGGACTTGCTCAGCAATTATTTATTAGTAAAAGCCAAGCCTAT
BLASTHIT_4          1 ---TCTTTAACATTAATTGGACTTGCCCAGCAATTATTTATTAGTAAAAGCCAAGCCTAT
BLASTHIT_5          1 -----------------------------GCAAGTATTT-TTAGTAAAA-CCAGGCCTAT
BLASTHIT_6          1 -------------------------------AATTATTTATTAGAAAAAG-CAACCCTAT
BLASTHIT_7          1 ------------------------------------------AGTAAAAGCCAAGCCCTT
BLASTHIT_8          1 ----CTTTAACATTAATTGGACTTCTTCAGCAA---------------------------
BLASTHIT_9          1 ----CTTTAACATTAACTGGACTTCCTCAGCAA---------------------------
BLASTHIT_10         1 ----CTTTAACATTAATTGGACTTCTTCAGCAA---------------------------
BLASTHIT_11         1 ---------------------------------TTGTTTATTAGTAAAAGTAAGGCTTAT
BLASTHIT_12         1 ----------------------------AGCGATTATTTTTTAGTAAAAGCAAAGCC---
BLASTHIT_13         1 --------------------------------------TATTAGAAAAAGCCAAGCCTAT
BLASTHIT_14         1 ---------------------------------TTACTTATTTAGTAAAAGCCAAGCTCT
BLASTHIT_15         1 ---------------------------------------ATTAGTAAAAGCCATGCCTAT
BLASTHIT_16         1 ---------------------------------TTGTTTATTAGTAAAAGTAAGGCTTAT
BLASTHIT_17         1 -------------------------------AATGATTTATTAGTAAAAGCCAAACC---
BLASTHIT_18         1 ---------------------------------------ATTAGTAAAAGCCAAGCCTAT
BLASTHIT_19         1 ------------------GGACTTG-GCAGCAATTATTTATTCTTCAAAGC--AGACTAT
BLASTHIT_20         1 --------------------------------ATTATTTATTAGTAAAAGCCA-------
...                 1 ------------------------------------------------------------
...                 1 ------------------------------------------------------------
N=206               1 ------------------------------------------------------------

Ecfaeca_g3060_P    39 AAATTAAGAAATCA
query_g3060_1      61 AAATTAAGAAATCA
BLASTHIT_1         61 AAATTAAGAAATCA
BLASTHIT_2         61 AAATTAAGAAATCA
BLASTHIT_3         61 AAATTAAGAAATCA
BLASTHIT_4         58 AAATTAAGAAATCA
BLASTHIT_5         30 AAATTA--------
BLASTHIT_6         29 AAA-----------
BLASTHIT_7         19 AATTTAAGAGATCA
BLASTHIT_8            --------------
BLASTHIT_9            --------------
BLASTHIT_10           --------------
BLASTHIT_11        28 GAACTAAGAAAT--
BLASTHIT_12           --------------
BLASTHIT_13        23 ATA-TAAGA-----
BLASTHIT_14        28 ATAAAT--------
BLASTHIT_15        22 AAA-----------
BLASTHIT_16        28 GAACTAAGAAAT--
BLASTHIT_17           --------------
BLASTHIT_18        22 --------------
BLASTHIT_19        40 AA------------
BLASTHIT_20           --------------
...                   --------------
...                   --------------
N=206                 --------------
```

BLAST HIT	Species	Query coverage [%]	Identity [%]	Accession	redundant hits
1	Enterococcus faecalis	100	99	NC_004668.1	687 x
2	Enterococcus sp.	100	99	NZ_JWBQ01000066.1	2 x
3	Enterococcus faecalis	100	99	NZ_GL454749.1	-
4	Enterococcus faecalis	96	99	NZ_PTYL01000009.1	-
5	Flavobacterium sp.	48	87	NZ_CP031557.1	-
6	Buchnera aphidicola	42	90	NC_004061.1	1 x
7	Acinetobacter johnsonii	43	88	NZ_FUUY01000018.1	-
8	Pasteurella multocida subsp. septica	39	90	NZ_UGSW01000001.1	1 x
9	Pasteurella multocida	39	90	NZ_PSQH01000001.1	4 x
10	Pasteurella multocida	39	90	NZ_CP023972.1	-
11	Enterococcus thailandicus	53	82	NZ_LWMN01000016.1	2 x
12	Algoriphagus halophilus	39	90	NZ_FSRC01000001.1	-
13	Candidatus Borrelia	41	89	NZ_CP025785.1	-
14	Elizabethkingia ursingii	43	89	NZ_MBDS01000002.1	-
15	Clostridiales bacterium	32	96	NZ_JQKL01000001.1	-
16	Enterococcus sp.	53	82	NZ_NGMR01000001.1	-
17	Flavobacterium beibuense	35	92	NZ_JRLV01000001.1	-
18	Aliterella atlantica	28	100	NZ_JYON01000010.1	-
19	Herpetosiphon geysericola	56	79	NZ_LGKP01000017.1	-
20	Corynebacterium kutscheri	28	100	NZ_CP011312.1	-

* anomalous sequences excluded from alignment
gi|1005608163|ref|NZ_FFHQ01000001.1|Listeria monocytogenes strain 2842STDY5753961
gi|522836690|ref|NZ_KE352316.1|Enterococcus faecalis strain SB2C-2 Scaffold89
gi|1120479204|ref|NZ_FQTZ01000012.1|Enterococcus faecium isolate Hp_76-17_S13
gi|1129541613|ref|NZ_CWML01000029.1|Listeria monocytogenes isolate LM52

Ec_faeci_cysS_3_P1

```
Ecfaeci_cysS_P1     1 GCAGCCACCAATTTACAACGA---------------------------------------
query_cysS_3        1 GCAGCCACCAATTTACAACGANTGAAGAATACGTTTGACAATTTGAANTTCCGCCAAGAA
BLASTHIT_1          1 GCAGCCACCAATTTACAACGACTGAAGAATACGTTTGACAATTTGAAATTCCGCCAAGAA
BLASTHIT_2          1 GCAGCCACCAATTTACAACGACTGAAGAATACGTTTGACAATTTGAAATTCCGCCAAGAA
BLASTHIT_3          1 GCAGCCACCAATTTACAACGACTGAAGAATACGTTTGACAATTTGAAGTTCCGCCAAGAA
BLASTHIT_4          1 GCAGCCACCAATTTACAACGATTGAAGAATACGTTTGACAATTTGAAATTCCGCCAAGAA
BLASTHIT_5          1 GCAGCCACCAATTTACAACGACTGAAGAATACGTTTGACAATTTGAAATTCCGCCCAGAA
BLASTHIT_6          1 GCAGCCACCAATTTACAACGACTGAAGAATACGTTTGACAATTTGAAATTCTGCCAAGAA
BLASTHIT_7          1 GCAGCCACCAATTTACAACGACTGAAGAATACGTTTGACAATTTGAAATTCCGCCAAGAA
BLASTHIT_8          1 GCAGCCACCAATTTACAACGACTGAAGAATACGTTTGACAATTTGAAATTCCGCCAAGAA
BLASTHIT_9          1 GCAGCCACCAATTTACAACGACTGAAGAATACGGTTGACAATTTGAAATTCCGCCAAGAA
BLASTHIT_10         1 GCAGCGACAAACTTACAACGATTGAAAAATACGTTTGATAATCTAAACTTCCG-------
BLASTHIT_11         1 GCAGCGACAAATTTACAACGCTTGAAAAATACGTTTGATAATCTAAACTTCCG-------
BLASTHIT_12         1 GCAGCGACAAACTTACAGCGATTGAAAAATACGTTTGATAATCTAAACTTCCG-------
BLASTHIT_13         1 GCAGCGACAAACTTACAACGATTGAAAAATACGTTTGATAATCTACACTTCCG-------
BLASTHIT_14         1 GCAGCGACAAACTTACAGCGATTGAAAAATACGTTTGATAATCTAAACTTCCG-------
BLASTHIT_15         1 GCAGCAATTAATTTACAACGATTGAGAAATACTTTTGAAAATCTTAAATTCCG-------
BLASTHIT_16         1 GCAGCGACAAACTTGCAACGTTTAAAAAATACGTTTGAAAACTTAAAATTCCG-------
BLASTHIT_17         1 -------CCAATTTCCAACGATTGAAGAATACG---------------------------
BLASTHIT_18         1 -------------------------------------------------------AAGAA
BLASTHIT_19         1 ----------------------TGAAGAATACGTTTGACAATGTTCAAATTC--------
BLASTHIT_20         1 -----------------------------------------------------------A
...                 1 ------------------------------------------------------------
...                 1 ------------------------------------------------------------
N=44                1 ------------------------------------------------------------

Ecfaeci_cysS_P1    22 ----CCTCAGAGAATTTGGCAGATGA
query_cysS_3       61 ACTGCCTCAGAGAATTTGGCAGATGA
BLASTHIT_1         61 ACTGCCTCAGAGAATTTGGCAGATGA
BLASTHIT_2         61 ACTGCCTCAGAGAATTTGGCAGATGA
BLASTHIT_3         61 ACTGCCTCAGAGAATTTGGCAGATGA
BLASTHIT_4         61 ACTGCCTCAGAGAATTTGGCAGATGA
BLASTHIT_5         61 ACTGCCTCAGAGAATTTGGCAGATGA
BLASTHIT_6         61 ACTGCCTCAGAGAATTTGGCAGATGA
BLASTHIT_7         61 ACTGCCTCAGATAATTTGGCAGATGA
BLASTHIT_8         61 ATTGCCTCAGAGAATTTGGCAGATGA
BLASTHIT_9         61 ACTGCCTCAGAGAATTTGGCAGATGA
BLASTHIT_10           --------------------------
BLASTHIT_11           --------------------------
BLASTHIT_12           --------------------------
BLASTHIT_13           --------------------------
BLASTHIT_14           --------------------------
BLASTHIT_15           --------------------------
BLASTHIT_16           --------------------------
BLASTHIT_17           --------------------------
BLASTHIT_18         6 ACTGCCTCAGCGAATTTGG-------
BLASTHIT_19           --------------------------
BLASTHIT_20         2 ACTGCCTCAGAGAATTTGGC------
...                   --------------------------
...                   --------------------------
N=44                  --------------------------
```

BLAST HIT	Species	Query coverage [%]	Identity [%]	Accession	redundant hits
1	Enterococcus faecium	100	98	NC_017960.1	1051 x
2	Enterococcus sp.	100	98	NZ_AJRB03000003.1	37 x
3	Enterococcus faecium	100	98	NZ_PTVC01000111.1	-
4	Enterococcus faecium	100	98	NZ_KB029683.1	-
5	Enterococcus sp.	100	97	NZ_KV829502.1	-
6	Enterococcus faecium	100	97	NZ_PUAH01000015.1	-
7	Enterococcus faecium	100	97	NZ_PUBG01000061.1	-
8	Enterococcus faecium	100	97	NZ_PTTS01000223.1	-
9	Enterococcus faecium	100	97	NZ_MJDZ01000007.1	-
10	Enterococcus mundtii	62	83	NZ_PTUS01000014.1	17 x
11	Enterococcus mundtii	62	83	NZ_PYGS01000082.1	-
12	Enterococcus mundtii	62	81	NC_022878.1	-
13	Enterococcus mundtii	62	81	NZ_AFWZ01000346.1	-
14	Enterococcus pernyi	62	81	NZ_KV388085.1	-
15	Enterococcus ratti	62	79	NZ_JXLB01000002.1	-
16	Enterococcus villorum	62	77	NZ_MJEB01000016.1	1 x
17	Bacillus okhensis	30	92	NZ_JRJU01000050.1	-
18	Anaplasma phagocytophilum	28	96	NZ_LANV01000001.1	17 x
19	Chitinophaga terrae	34	89	NZ_FNRL01000002.1	-
20	Amycolatopsis sacchari	24	100	NZ_FORP01000012.1	-

Ec_faeci_purD_2_P0

```
Ecfaeci_purD_P0     1 GGAATGAAAGAACTAGGACGCT-----------------------------ATTGCTACG
query_purD_2        1 GGAATGAAAGAACTAGGACGCTCTTTCACGGGTGTTTTATATGCGGGTTTNATTGCTACG
BLASTHIT_1          1 GGAATGAAAGAACTAGGACGCTCTTTCACGGGTGTTTTATATGCGGGTTTGATTGCTACG
BLASTHIT_2          1 GGAATGAAAGAACTAGGACGCTCTTTCACGGGTGTTTTATATGCGGGTTTGATTGCTACG
BLASTHIT_3          1 GGAATGAAAGAACTAGGACGCTCTTTCACGGGTGTTTTATATGCGGGTTTAATTGCTACG
BLASTHIT_4          1 GGAATGAAAGAACTAGGACGCTCTTTCACGGGTGTTTTATATGCGGGTTTAATTGCTACG
BLASTHIT_5          1 GGAATGAAAGAACTAGGACGCTCTTTCACAGGTGTTTTATATGCGGGTTTGATTGCTACG
BLASTHIT_6          1 ---------------GGGCGTTCTTTCACGGGTGTTTTATATGCAGGGTTAATTGCTACG
BLASTHIT_7          1 GGAATGAAGGAACTAGGACGTCCGTTTACTGGTATTTTATATGCAGGGTTAATTGCCACA
BLASTHIT_8          1 GGAATGAAGGAACTAGGACGTCCATTTACTGGTATTTTATATGCAGGGTTAATTGCCACA
BLASTHIT_9          1 GGAATGAAAGAGCTAGGACGTCCATTTACTGGTATTTTATATGCAGGGTTAATTGCCACA
BLASTHIT_10         1 ---------------GGTCGCTCTTTTACAGGCATTTTATATGCAGGTTTAATTGCTACG
BLASTHIT_11         1 ---------------GGTCGCTCTTTTACAGGCATTTTATATGCAGGTTTAATTGCTACG
BLASTHIT_12         1 ---------------GGACGCTCTTTCACAGGCATTTTATATGCTGGCTTAATTGCCACG
BLASTHIT_13         1 GGAATGAAAGAGATAGGTCGTTCATTTACTGGTATTTTATATGCAGGGTTGATTGCCAC-
BLASTHIT_14         1 GGAATGAAAGAGATAGGTCGTTCATTTACTGGTATTTTATATGCAGGGCTGATTGCCACG
BLASTHIT_15         1 --------------AGGTCGTTCGTTTACTGGTGTTTTATATGCCGGATTGATTGCTACG
BLASTHIT_16         1 GGAATGATTGATCAAGGAACACCCTTT-ACGGGTATTTTATATGCGGGGCTGATTGCTAC
BLASTHIT_17         1 ---ATGGAAGAAGAAGG-CGCATCCTTCACTGGAGTTTTATATGCAGGATTAATTCTTAC
BLASTHIT_18         1 GGAATGAAAGAGATAGGTCGTTCATTTACTGGTATTTTATATGCAGGACTGATTGCCAC-
BLASTHIT_19         1 GGAATGAAAGAGATAGGTCGTTCATTTACTGGTATTTTATATGCAGGGCTGATTGCCAC-
BLASTHIT_20         1 --AATGGAAGAAGAAGGCGTCTCTTTCACAGGGATATTATATGCGGGCTTAATTCTTACA
...                 1 ------------------------------------------------------------
...                 1 ------------------------------------------------------------
N=67                1 ------------------------------------------------------------

Ecfaeci_purD_P0    32 AAGGAAGGACC-
query_purD_2       61 AAGGAAGGACC-
BLASTHIT_1         61 AAGGAAGGACC-
BLASTHIT_2         61 AAGGAAGGACC-
BLASTHIT_3         61 AAGGAAGGACC-
BLASTHIT_4         61 AAGGAAGGACC-
BLASTHIT_5         61 AAGGAAGGACC-
BLASTHIT_6         46 AATAAAGG----
BLASTHIT_7         61 GCAGAAGGACC-
BLASTHIT_8         61 GCAGAAGGACC-
BLASTHIT_9         61 GCAGAAGGACC-
BLASTHIT_10        46 AATAAAGGA---
BLASTHIT_11        46 AATAAAGGA---
BLASTHIT_12        46 AA----------
BLASTHIT_13           ------------
BLASTHIT_14        61 GCAGAAGGGCC-
BLASTHIT_15        47 AA----------
BLASTHIT_16        60 AGTTGAAGGACC
BLASTHIT_17        57 GAAAGAAGGTCC
BLASTHIT_18           ------------
BLASTHIT_19           ------------
BLASTHIT_20        59 AAAGAAGGGCC-
...                   ------------
...                   ------------
N=67                  ------------
```

BLAST HIT	Species	Query coverage [%]	Identity [%]	Accession	redundant hits
1	Enterococcus faecium	100	99	NC_017960.1	1080 x
2	Enterococcus sp.	100	99	NZ_AJRB03000044.1	37 x
3	Enterococcus sp.	100	99	NZ_NGMF01000001.1	-
4	Enterococcus faecium	100	99	NZ_JH804745.1	-
5	Enterococcus faecium	100	97	NZ_JWEB01000121.1	-
6	Bacillus cihuensis	75	87	NZ_AYSD01000010.1	-
7	Enterococcus mundtii	100	79	NZ_PYGS01000033.1	5 x
8	Enterococcus mundtii	100	79	NZ_FOUC01000005.1	-
9	Enterococcus mundtii	100	79	NZ_NGMS01000001.1	-
10	Paenibacillus sp.	76	83	NZ_LMVB01000002.1	-
11	Bacillus sp.	76	83	NZ_LMXG01000002.1	-
12	Bacillus loiseleuriae	66	85	NZ_LFZW01000001.1	-
13	Enterococcus pernyi	83	80	NZ_KV388085.1	-
14	Enterococcus mundtii	100	76	NZ_PTUS01000023.1	-
15	Enterococcus faecalis	68	83	NZ_KE351686.1	-
16	Enterococcus gallinarum	99	76	NZ_BCQE01000018.1	9 x
17	Ureibacillus thermosphaericus	95	76	NZ_AJIK01000018.1	1 x
18	Enterococcus mundtii	83	78	NZ_AFWZ01000136.1	-
19	Enterococcus mundtii	83	78	NZ_PYGU01000006.1	-
20	Lysinibacillus sp.	97	75	NZ_LT985980.1	-

* anomalous sequences excluded from alignment
gi|1129541523|ref|NZ_CWML01000013.1|Listeria monocytogenes isolate LM52

Pd_acidi_asnS_2_P0

```
Pdacidi_asnS_P0     1 GCCATCCACGAATTCCTCCA-------------------------------------ACT
query_asnS_2        1 GCCATCCACGAATTCCTCCAAAAGAANAACTTTGTCTACGTTAACACTCCGTTGATNACT
BLASTHIT_1          1 GCCATCCACGAATTCCTCCAAAAGAACAACTTTGTCTACGTTAACACTCCGTTGATTACT
BLASTHIT_2          1 GCCATCCACGAATTCCTCCAAAAGAATAACTTTGTCTACGTTAACACTCCGTTGATTACT
BLASTHIT_3          1 GCCATCCACGAATTCCTCCAAAAGAATAACTTTGTCTACGTTAACACTCCGTTGATCACT
BLASTHIT_4          1 ------CACGAATACCTACAACACAATGACTTTGTTTACGTTAACACCCCAATCATCACA
BLASTHIT_5          1 ----------AATTCTTACAAAGCAACAACTTTGTTTACGTTAACACACCGATCATCACC
BLASTHIT_6          1 ----------AATTCTTACAAAGCAACAACTTTGTTTACGTTAACACACCGATTATCACC
BLASTHIT_7          1 ----------AATTCTTACAAAGCAACAACTTTGTTTACGTTAACACACCAATCATCACT
BLASTHIT_8          1 ----------AATTCTTACAAAGTAACAACTTTGTTTACGTTAACACACCAATCATCACT
BLASTHIT_9          1 ------CACGAATACCTACAACACAATGACTTTGTTTACGTTAACACCCCAATCATCACA
BLASTHIT_10         1 ------CACGAATACCTACAACACAATGACTTCGTTTACGTTAACACCCCAATCATCACA
BLASTHIT_11         1 ------CATGAATACCTACAACACAATGACTTTGTTTACGTTAACACCCCAATCATCACA
BLASTHIT_12         1 ------CACGAATACCTACAACACAATGACTTCGTTTACGTTAACACACCAATCATCACA
BLASTHIT_13         1 GCAATACACAAATTCTTTCAGGAGAGAAACTTTGTCTATGTGCATACTCCCTTGATCACA
BLASTHIT_14         1 GCTATTCACGAATACCTCCAAAAGAATGATTTCGTCTATGTTCATACACCAATCATCACT
BLASTHIT_15         1 GCAATTCACAAATTTTTTCAGGAAAAGAACTTTGTCTACGTTCAAACTCCAATTATTACT
BLASTHIT_16         1 ------CATGAATACTTACAACACAACGACTTCGTTTACGTTAACACCCCAATCATCACA
BLASTHIT_17         1 GCAATACACAAATTCTTTCAGGAGAGAAACTTTGTCTATGTGCATACTCCCCTGATCACA
BLASTHIT_18         1 GCCATCCACAAGTTCTTCCAGGAGAACGGCTTTGTCTACGCCCACTCTCCGCTGATCAC-
BLASTHIT_19         1 ------CATGAATACTTACAACACAACGATTTCGTATACGTTAACACACCAATTATCACT
BLASTHIT_20         1 GCTATCCACAAGTTCTTCCAGGAGAACGGCTTTGTCTACGCTCACTCTCCGCTGATCAC-
...                 1 ------------------------------------------------------------
...                 1 ------------------------------------------------------------
N=128               1 ------------------------------------------------------------

Pdacidi_asnS_P0    24 TCTAGTGATACGGAAGGTGC
query_asnS_2       61 TCTAGTGATACGGAAGGTGC
BLASTHIT_1         61 TCTAGTGATACGGAAGGTGC
BLASTHIT_2         61 TCTAGTGATACGGAAGGTGC
BLASTHIT_3         61 TCTAGTGATACGGAAGGTGC
BLASTHIT_4         55 AGTAGTGATACTGAAGGTGC
BLASTHIT_5         51 AGCAGTGATACTGAAGGTGC
BLASTHIT_6         51 AGCAGTGATACTGAAGGTGC
BLASTHIT_7         51 AGCAGTGATACTGAAGGTGC
BLASTHIT_8         51 AGCAGTGATACTGAAGGTGC
BLASTHIT_9         55 AGTAGTGATACTGAAGGGGC
BLASTHIT_10        55 AGTAGTGATACTGAAGGTGC
BLASTHIT_11        55 AGTAGTGATACTGAAGGTGC
BLASTHIT_12        55 AGTAGTGATACTGAAGGTGC
BLASTHIT_13        61 GGAAGTGATGCAGAAGGTGC
BLASTHIT_14        61 AGTTCTGATGCCGAAGGTGC
BLASTHIT_15        61 GGAAGTGACGCGGAAGGTGC
BLASTHIT_16        55 AGTAGTGATACTGAAGGTGC
BLASTHIT_17        61 GGAAGTGATGCAGAAGGTGC
BLASTHIT_18           --------------------
BLASTHIT_19        55 AGCAGTGATACTGAAGGTGC
BLASTHIT_20           --------------------
...                   --------------------
...                   --------------------
N=128                 --------------------
```

BLAST HIT	Species	Query coverage [%]	Identity [%]	Accession	redundant hits
1	Pediococcus acidilactici	100.0	98.0	NZ_CP015206.1	16 x
2	Pediococcus acidilactici	100.0	98.0	NZ_QYRQ01000020.1	-
3	Pediococcus acidilactici	100.0	98.0	NZ_JQCC01000002.1	-
4	Lactobacillus kunkeei	92.0	78.0	NZ_CP012920.1	11 x
5	Pediococcus pentosaceus	88.0	79.0	NC_008525.1	11 x
6	Pediococcus pentosaceus	88.0	79.0	NZ_JDVW01000006.1	-
7	Pediococcus pentosaceus	88.0	79.0	NZ_JQBF01000008.1	-
8	Pediococcus pentosaceus	88.0	79.0	NC_022780.1	-
9	Lactobacillus kunkeei	92.0	77.0	NZ_JXCZ01000012.1	-
10	Lactobacillus kunkeei	92.0	77.0	NZ_JXDA01000004.1	-
11	Lactobacillus kunkeei	92.0	77.0	NZ_BDDX01000015.1	-
12	Lactobacillus kunkeei	92.0	77.0	NZ_JXDD01000007.1	-
13	Ruminiclostridium cellobioparum subsp. termitidis	100.0	74.0	NZ_AORV01000003.1	-
14	Lactobacillus sp.	100.0	74.0	NZ_CP031933.1	-
15	Leptotrichia sp.	100.0	74.0	NZ_CP016753.1	-
16	Lactobacillus kunkeei	92.0	74.0	NZ_JXDC01000005.1	-
17	Ruminiclostridium cellobioparum	100.0	72.0	NZ_JHYD01000026.1	-
18	Faecalibacterium prausnitzii	74.0	76.0	NZ_QVFB01000007.1	9 x
19	Lactobacillus sp.	92.0	73.0	NZ_CP032626.1	-
20	Faecalibacterium prausnitzii	74.0	76.0	NZ_NMTZ01000018.1	-

Pd_acidi_g1164_1_P0

```
Pdacidi_g1164_P    1 TTTAGGAGCAATCATCGCAATG---------------------------------GGTAG
query_g1164_1      1 TTTAGGAGCAATCATCGCAATGATTTTCGGCATTATCTGGGTCACGNNTGGCTTTGGTAG
BLASTHIT_1         1 TTTAGGAGCAATCATCGCAATGATTTTCGGCATTATCTGGGTCACGATTGGCTTTGGTAG
BLASTHIT_2         1 --------CAATCATCGCAATGATTTTGGGTTTTATCTG---------------------
BLASTHIT_3         1 TTCAGGAGCAATCATTGCCATTCTTTTCGGCATTA-------------------------
BLASTHIT_4         1 ----------------GCAGTGATTTTCGGCATCATCTGGGGCAC---------------
BLASTHIT_5         1 --------CAATCATCGCACTGATTATCGGGATTATC-----------------------
BLASTHIT_6         1 --------CAATCATCGCACTGATTATCGGGATTATC-----------------------
BLASTHIT_7         1 TTTAGAAGCAATCATCGCAATGAT------------------------------------
BLASTHIT_8         1 ----------ATCATCGAAATATTTTTCTGTATTTTCTGGGTCACG--------------
BLASTHIT_9         1 ---------------CGCAGTAATCTTCGGCATTATCTGGGGCACG--------------
BLASTHIT_10        1 TTTATGAGCAATCATCTCAATGATTT----------------------------------
BLASTHIT_11        1 TTTAGCAGCAATCATCGCAGTGATTT----------------------------------
BLASTHIT_12        1 TTTATGAGCAATCATCTCAATGATTT----------------------------------
BLASTHIT_13        1 TTTAGGAGCAATCATCGCTAATGATATT--------------------------------
BLASTHIT_14        1 TTTAGGAGCAATCATCGCTAATGATATT--------------------------------
BLASTHIT_15        1 ----------ATCATCGCAGTAATTTTAGTCATTATCTGGG-------------------
BLASTHIT_16        1 TTTAGCAGCAATCATCGCAGTGATTT----------------------------------
BLASTHIT_17        1 -------------ATCGCAATGATTTTCGGCATT--------------------------
BLASTHIT_18        1 -------GCAATCATCGCAATGATTTTC--------------------------------
BLASTHIT_19        1 -----------------------TTTTCGGCATTATCTGGGTCA----------------
BLASTHIT_20        1 ------------CATCGAATTGATTTTCGGCATTATCT----------------------
...                1 ------------------------------------------------------------
...                1 ------------------------------------------------------------
N=207              1 ------------------------------------------------------------

Pdacidi_g1164_P   28 CGCGGTATTAGTTGG
query_g1164_1     61 CGCGGTATTAGTTGG
BLASTHIT_1        61 CGCGGTATTAGTTGG
BLASTHIT_2           ---------------
BLASTHIT_3           ---------------
BLASTHIT_4           ---------------
BLASTHIT_5           ---------------
BLASTHIT_6           ---------------
BLASTHIT_7           ---------------
BLASTHIT_8           ---------------
BLASTHIT_9           ---------------
BLASTHIT_10          ---------------
BLASTHIT_11          ---------------
BLASTHIT_12          ---------------
BLASTHIT_13          ---------------
BLASTHIT_14          ---------------
BLASTHIT_15          ---------------
BLASTHIT_16          ---------------
BLASTHIT_17          ---------------
BLASTHIT_18          ---------------
BLASTHIT_19          ---------------
BLASTHIT_20          ---------------
...                  ---------------
...                  ---------------
N=207                ---------------
```

BLAST HIT	Species	Query coverage [%]	Identity [%]	Accession	redundant hits
1	Pediococcus acidilactici	100.0	97.0	NZ_CP015206.1	22 x
2	Helicobacter sp.	41.0	90.0	NZ_MLAM01000013.1	-
3	Gallibacterium genomosp.	47.0	86.0	NZ_JTJS01000032.1	-
4	Erwinia iniecta	39.0	90.0	NZ_JRXE01000016.1	1 x
5	Yersinia kristensenii	39.0	90.0	NZ_CWJK01000005.1	4 x
6	Yersinia frederiksenii	39.0	90.0	NZ_CP009364.1	1 x
7	Bacillus vietnamensis	32.0	96.0	NZ_LIXZ01000003.1	-
8	Catellicoccus marimammalium	48.0	83.0	NZ_AMYT01000022.1	-
9	Erwinia persicina	41.0	87.0	NZ_BCTN01000009.1	1 x
10	Megamonas rupellensis	35.0	92.0	NZ_KB899597.1	3 x
11	Acinetobacter kyonggiensis	35.0	92.0	NZ_FNPK01000003.1	-
12	Megamonas sp.	35.0	92.0	NZ_FOCY01000013.1	-
13	Macrococcus epidermidis	36.0	92.0	NZ_PZJH01000003.1	-
14	Macrococcus goetzii	36.0	92.0	NZ_MJBI02000002.1	-
15	Sphingobacterium faecium	41.0	87.0	NZ_QBKH01000003.1	-
16	Acinetobacter sp.	35.0	92.0	NZ_NEGM01000001.1	1 x
17	Bacillus sp.	28.0	100.0	NZ_LMTJ01000002.1	9 x
18	Pseudomonas batumici	28.0	100.0	NZ_JXDG01000015.1	-
19	Rhodovulum sp.	28.0	100.0	NZ_QPLK01000071.1	-
20	Roseovarius sp.	35.0	92.0	NZ_OUMZ01000007.1	-

* anomalous sequences excluded from alignment
gi|522836680|ref|NZ_KE352306.1|Enterococcus faecalis strain SB2C-2 Scaffold825

Pd_pento_nagK_1_P0

```
Pdpento_nagK_P0    1 GGGTGTCACTAACGGAGCAA----------------------------------------
query_nagK_1       1 GGGTGTCACTAACGGAGCAACNATCGCCCTTGATGATGGTGTAAGTTCTGGCTTAGTTTT
BLASTHIT_1         1 GGGTGTCACTAACGGAGCAACCATCGCCCTTGATGATGGTGTAAGTTCTGGCTTAGTTTT
BLASTHIT_2         1 -------------------------------------GGCGTAAGTTCAGGAATTGTTTT
BLASTHIT_3         1 ----------------------------------------------TCTCGCTTAGTTTT
BLASTHIT_4         1 ------------------------------------------AAGTTATGGCTTAGTTTT
BLASTHIT_5         1 ------------------------------------------------------------
BLASTHIT_6         1 ---------------------------------------------TTCTTGTTTACTTTT
BLASTHIT_7         1 ----------------------------------------------------TTATTTTT
BLASTHIT_8         1 ---------------------------------------------TTCTTGTTTACTTTT
BLASTHIT_9         1 ----------------------ATCTCCCTCGATGATGGTGTAAGTTTGGGCTT------
BLASTHIT_10        1 ----------------------ATCGCCCTTGATTATGATTTAAGTTATGG---AGTTTT
BLASTHIT_11        1 ---------------------------------------------TTCTGGCTTAGTTTT
BLASTHIT_12        1 ----------------------ATCGCCCTTGATTATGATTTAAGTTATGG---AGTTTT
BLASTHIT_13        1 -----------------------------------------------CTGGCTTACTTTT
BLASTHIT_14        1 -----------------------------------------------CTGGCTTACTTTT
BLASTHIT_15        1 ------------------------------------------------------------
BLASTHIT_16        1 -----------------------------------------------CTGGTTTAGTTAT
BLASTHIT_17        1 ------------------------------TGATGATGGTGTAATTTCTGGATT-GTCTT
BLASTHIT_18        1 ----------------------------------------------------TTAGTTTT
BLASTHIT_19        1 --------------------------------------------------------TTAT
BLASTHIT_20        1 ------------------------------TGATGATGTTGTAGGTTCTGGCTTAG----
...                1 ------------------------------------------------------------
...                1 ------------------------------------------------------------
N=114              1 ------------------------------------------------------------

Pdpento_nagK_P0   21 ---------------------------CTTACAAGCGGGCGGAATTG
query_nagK_1      61 AAATCACCAGATTTTTTCTGGTTCCCACTTACAAGCGGGCGGAATTG
BLASTHIT_1        61 AAATCACCAGATTTTTTCTGGTTCCCACTTACAAGCGGGCGGAATTG
BLASTHIT_2        24 AAACAACAAAATCTTATCAGGCTCTCACTTACAAGCCGGTGAAGTTG
BLASTHIT_3        15 AAATAGCCCGATTTTTTCT----------------------------
BLASTHIT_4        19 AAATAA--AGATTTTT-------------------------------
BLASTHIT_5         1 -------CAGATTTTTTTTGGTTCCCACTTGCAA-------------
BLASTHIT_6        16 AAATCACCAGATTTATT------------------------------
BLASTHIT_7         9 AAATCATCAGATTTTTTCT----------------------------
BLASTHIT_8        16 AAATCACCAGATTTATT------------------------------
BLASTHIT_9           -----------------------------------------------
BLASTHIT_10       36 AA---------------------------------------------
BLASTHIT_11       16 CAATAACTTCCAGATTTTTT---------------------------
BLASTHIT_12       36 AA---------------------------------------------
BLASTHIT_13       14 AAATCACCAGA------------------------------------
BLASTHIT_14       14 AAATCACCAGA------------------------------------
BLASTHIT_15        1 -----------TTTTTTCTGGTTCCCACTTATAAACG----------
BLASTHIT_16       14 TAACAACCAAATTTTTTCTGGTT------------------------
BLASTHIT_17       30 AAA--------------------------------------------
BLASTHIT_18        9 ATTAATTACCAGATTTTTCCTGACTTCCACTT---------------
BLASTHIT_19        5 AAATCACCAGATCTTTTCTGTTGCCCA--------------------
BLASTHIT_20          -----------------------------------------------
...                  -----------------------------------------------
...                  -----------------------------------------------
N=114                -----------------------------------------------
```

BLAST HIT	Species	Query coverage [%]	Identity [%]	Accession	redundant hits
1	Pediococcus pentosaceus	100.0	99.0	NC_008525.1	14 x
2	Pediococcus stilesii	65.0	74.0	NZ_JQBX01000011.1	-
3	Zhouia amylolytica	31.0	88.0	NZ_FPAG01000004.1	-
4	Clostridium perfringens	30.0	86.0	NZ_CYYX01000001.1	4 x
5	Flavobacterium haoranii	25.0	93.0	NZ_FQZH01000005.1	-
6	Acinetobacter bereziniae	30.0	88.0	NZ_QWFV01000007.1	3 x
7	Mycoplasma auris	25.0	93.0	NZ_QKUB01000015.1	-
8	Acinetobacter sp.	30.0	88.0	NZ_AMFQ01000033.1	4 x
9	Thiomonas intermedia	30.0	88.0	NC_014153.1	-
10	Leptospira meyeri	34.0	80.0	NZ_AKXE01000002.1	1 x
11	Chryseobacterium scophthalmum	30.0	83.0	NZ_FSRQ01000001.1	-
12	Leptospira sp.	34.0	80.0	NZ_NPEB01000007.1	2 x
13	Acinetobacter brisouii	22.0	96.0	NZ_JZRE01000019.1	-
14	Acinetobacter sp.	22.0	96.0	NZ_KB851219.1	-
15	Clostridium pasteurianum	24.0	92.0	NC_021182.1	-
16	Mycoplasma pullorum	34.0	83.0	NZ_CP017813.1	1 x
17	Lutibacter profundi	30.0	87.0	NZ_CP013355.1	-
18	'Nostoc azollae'	35.0	80.0	NC_014248.1	-
19	Elizabethkingia meningoseptica	29.0	87.0	NZ_BARD01000013.1	19 x
20	Sporomusa silvacetica	24.0	92.0	NZ_LSLK01000054.1	-

Pd_pento_g4364_1_P0

```
Pdpento_g4364_P    1 TGCTTACCACCAACCTGCTT------------------------------------ACGG
query_g4364_1      1 TGCTTACCACCAACCTGCTTATAACCAAGCTNGTCAAAAGATAACGGTTAAATTTAACGG
BLASTHIT_1         1 TGCTTACCACCAACCTGCTTATAACCAAGCTAGTCAAAAGATAACGGTTAAATTTAACGG
BLASTHIT_2         1 TGCTTACCACCAACCTGCTTATAACCAAGCTGGTCAAAAGATAACGGTTAAATTTAACGG
BLASTHIT_3         1 -----------------------------------AAAAGATAACGGTTAATGTTTTAAC
BLASTHIT_4         1 -------CACCAACCTGCTCATAACCAAGCT-----------------------------
BLASTHIT_5         1 TGCTTGTCACAAACCTGCTTATAACCAAG-------------------------------
BLASTHIT_6         1 ----------CAACCTGCTCATAACCAAGCTCGTCA------------------------
BLASTHIT_7         1 --------------------------------GTCGAAAGA--ACGGTTAAATTTAACGG
BLASTHIT_8         1 ----------------------------------------------GTTAAATCTAACGG
BLASTHIT_9         1 --------------------------AAGATGGTCAAAAGATAACGGTTAA---------
BLASTHIT_10        1 ------------------------------------------AACGGTTAAATTTAACAG
BLASTHIT_11        1 -----------------------------------AAAAGATAACGTTTCAATTAAACGG
BLASTHIT_12        1 TGCTTACCACCAACGTGCTTATCAGCAA--------------------------------
BLASTHIT_13        1 -----------------------------------------TAACGGTTAATTTTAACGG
BLASTHIT_14        1 ------------------------------------AAAAATAACGGATAAATTTAACGA
BLASTHIT_15        1 --------------------------------GTCAAAAGATTACGGTTAAA-TTGACGG
BLASTHIT_16        1 --------ACCAACCTGCTTCTAACCAAGCT-----------------------------
BLASTHIT_17        1 ---------------------------------------------GGTTAAATTTAACCG
BLASTHIT_18        1 -----------------CTTAACATCAAGCTATTTAAAAGAAAAGCGGTTAAATTTCATG
BLASTHIT_19        1 --------------------------------GTCAAAAGATTACGGTTAAA-TTGACGG
BLASTHIT_20        1 -----------------------------------------TAACGGTTAATTTTAACGG
...                1 ------------------------------------------------------------
...                1 ------------------------------------------------------------
N=37               1 ------------------------------------------------------------

Pdpento_g4364_P   25 AAACCTAGAACGTCCA
query_g4364_1     61 AAACCTAGAACGTCCA
BLASTHIT_1        61 AAACCTAGAACGTCCA
BLASTHIT_2        61 AAACCTAGAACGTCCA
BLASTHIT_3        26 GGAAAC----------
BLASTHIT_4           ----------------
BLASTHIT_5           ----------------
BLASTHIT_6           ----------------
BLASTHIT_7        27 AA--------------
BLASTHIT_8        15 AAACCTAGA-------
BLASTHIT_9           ----------------
BLASTHIT_10       19 AACTACCGAGAACG--
BLASTHIT_11       26 AAA-------------
BLASTHIT_12          ----------------
BLASTHIT_13       20 AAAC------------
BLASTHIT_14       25 AAAGCCAGA-------
BLASTHIT_15       28 AA--------------
BLASTHIT_16          ----------------
BLASTHIT_17       16 AAACCTAG--------
BLASTHIT_18       44 GAAA------------
BLASTHIT_19       28 AA--------------
BLASTHIT_20       20 AAAC------------
...                  ----------------
...                  ----------------
N=37                 ----------------
```

BLAST HIT	Species	Query coverage [%]	Identity [%]	Accession	redundant hits
1	Pediococcus pentosaceus	100.0	99.0	NC_008525.1	13 x
2	Pediococcus pentosaceus	100.0	99.0	NZ_JDVW01000004.1	-
3	Firmicutes bacterium	39.0	88.0	NZ_QUDS01000002.1	-
4	Pseudovibrio hongkongensis	32.0	96.0	NZ_LLWC01000022.1	-
5	Luteibacter rhizovicinus	38.0	90.0	NZ_JPLB01000036.1	1 x
6	Burkholderia multivorans	34.0	92.0	NZ_PVGD01000058.1	-
7	Streptococcus gallolyticus	37.0	88.0	NZ_FNFJ01000002.1	1 x
8	Unicellular cyanobacterium	30.0	96.0	NZ_NETF01000197.1	-
9	Staphylococcus gallinarum	33.0	92.0	NZ_JXCF01000010.1	1 x
10	Pyrinomonas methylaliphatogenes	40.0	86.0	NZ_CBXV010000006.1	-
11	Maribacter sedimenticola	37.0	89.0	NZ_FZNV01000002.1	-
12	Mannheimia haemolytica	37.0	89.0	NC_021082.1	66 x
13	Hungateiclostridium thermocellum	30.0	96.0	NC_009012.1	6 x
14	Virgibacillus siamensis	43.0	85.0	NZ_FUIH01000007.1	-
15	Streptococcus sanguinis	38.0	89.0	NZ_GL890988.1	-
16	Bacillus sp.	30.0	96.0	NZ_QPJG01000005.1	-
17	Bacillus taeanensis	30.0	96.0	NZ_QOCW01000025.1	-
18	Actinobacillus minor	61.0	78.0	NZ_ACQL01000172.1	-
19	Streptococcus sp.	38.0	89.0	NZ_LSJN01000035.1	-
20	Ruminiclostridium thermocellum	30.0	96.0	NZ_CP016502.1	1 x

*Anomalous sequences excluded from alignment**

**gi|522836524|ref|NZ_KE352150.1|Enterococcus faecalis strain SB2C-2 Scaffold11**<br>**gi|522836690|ref|NZ_KE352316.1|Enterococcus faecalis strain SB2C-2 Scaffold89**<br>**gi|522836680|ref|NZ_KE352306.1|Enterococcus faecalis strain SB2C-2 Scaffold825**<br><br>**Excluded because we suspected a wrong taxonomic classification.**<br><br>The classification was corrected on 2018-10-26.<br>Enterococcus faecium SB2C-2 before 2018-10-26 (see Taxonomic-Update-Statistics)
**gi|1120478950|ref|NZ_FQTZ01000002.1|Enterococcus faecium isolate Hp_76-17_S13_**<br>**gi|1120479204|ref|NZ_FQTZ01000012.1|Enterococcus faecium isolate Hp_76-17_S13_**<br><br>**Excluded because we suspect a wrong taxonomic classification.**<br><br>Taxonomy report of BLAST search (using default values, Nucleotide collection) of this sequence (NZ_FQTZ01000002.1) shows best hits for Enterococcus faecalis and no hits for Enterococcus faecium.<br><br>BLAST search (using default values, Nucleotide collection) of this sequence (NZ_FQTZ01000012.1) shows best hits for Enterococcus faecalis and less similarity for Enterococcus faecium genome assemblies.
**gi|1423449487|ref|NZ_UEMU01000447.1|Escherichia coli strain KCRI-77E isolate RDK43_77E**<br><br>**Excluded because we suspect a contamination.**<br><br>Taxonomy report of BLAST search (using default values, Nucleotide collection) of this sequence (NZ_UEMU01000447.1) shows only results for Enterococcus faecalis.
**gi|1129541523|ref|NZ_CWML01000013.1|Listeria monocytogenes isolate LM52**<br>**gi|1129541613|ref|NZ_CWML01000029.1|Listeria monocytogenes isolate LM52**<br><br>**Excluded because we suspect a wrong taxonomic classification.**<br><br>The corresponding assembly (GCA_001495195.1) shows only 79.251 % gapped identity to the closest related Listeria monocytogenes assembly.<br><br>BLAST search (using default values, Nucleotide collection) of these sequences (NZ_CWML01000013.1, NZ_CWML01000029.1) shows high similarity with Enterococcus thailandicus and some similarity to other Enterococcus species but no hits for Listeria species.
**gi|1005608163|ref|NZ_FFHQ01000001.1|Listeria monocytogenes strain 2842STDY5753961**<br><br>**Excluded because we suspect a wrong taxonomic classification.**<br><br>The corresponding assembly (GCA_900017475.1) shows only 87.1677 % gapped identity to the closest related Listeria monocytogenes assembly.<br><br>Taxonomy report of BLAST search (using default values, Nucleotide collection) of the first 100000 bases of this sequence (NZ_FFHQ01000001.1) shows best hits for Enterococcus faecalis and no hits for Listeria species.
