# Supplementary material for: SpeciesPrimer: a bioinformatics pipeline dedicated to the design of qPCR primers for the quantification of bacterial species
Source: PeerJ. 2020 Feb 18;8:e8544. doi: 10.7717/peerj.8544 (PMC7034379; doi:10.7717/peerj.8544)
Supplement: Table S1 [file peerj-08-8544-s008.docx]

| **Tools** | **Features** | **Pipeline task** |
| --- | --- | --- |
| NCBI Entrez module (Biopython) | Offers a variety of tools and code to access NCBI data | Search and download of genome assemblies |
| BLAST+ | Compares sequences and finds regions of similarity in sequence databases | Assess the similarity of input sequences to sequences in the nt database |
| Prokka | Fast whole genome annotation for bacterial, archeal and viral genomes | Annotation of bacterial input genome assemblies |
| Roary | Fast pan genome pipeline working on standard desktop PC’s | Identify single copy core genes of the target species. |
| FastTree 2 | Fast approximately-maximum-likelihood phylogenetic trees from nucleotide or protein alignments | Phylogenetic tree of core gene alignments, can be used to assess if the input genomes are closely-related |
| Prank | Probabilistic multiple alignment program aiming at an evolutionarily correct alignment of closely-related sequences | Alignment of single copy core genes |
| consambig  (EMBOSS) | Creates an ambiguous consensus sequence from a multiple alignment | Build a consensus sequence to identify conserved sequences |
| GNU parallel | Shell tool for executing jobs in parallel from a input list | Run jobs (Prank, consambig) in parallel |
| Primer3 | Widely used program for the design of PCR primers offering many different input parameters | Design of PCR primers for target sequences |
| MFEprimer-2.0 | Fast primer quality control tool to assess PCR primer specificity | Assess the binding of primer sequences to target and non-target sequences |
| MPprimer | Design and quality control of multiplex PCR primers | Assess the stability of primer dimers |
| Mfold | Predicts secondary structures of single stranded nucleic acids | Assess the stability of secondary structures in PCR amplicons |
