# Supplementary material for: SpeciesPrimer: a bioinformatics pipeline dedicated to the design of qPCR primers for the quantification of bacterial species
Source: PeerJ. 2020 Feb 18;8:e8544. doi: 10.7717/peerj.8544 (PMC7034379; doi:10.7717/peerj.8544)
Supplement: Table S2 [file peerj-08-8544-s009.docx]

| **Target species** | **Gene** | **Primer** | **Sequence 5’-3’** | **Amplicon**  **size (bp)** |
| --- | --- | --- | --- | --- |
| ***E. faecalis*** | *acuI* | Ecfaeca_acuI-F | TCATTTCAAGCATTTACGTTAAGAGA | 73 |
|  |  | Ecfaeca_acuI-R | CGTCTAAAGTAATGGTTTCTAGTTGA |  |
|  | g3060 | Ecfaeca_g3060-F | CCCTCTTTAACATTAATTGGACTTGC | 74 |
|  |  | Ecfaeca_g3060-R | TGATTTCTTAATTTATAGGCTTGGCT |  |
| ***E. faecium*** | *cysS* | Ecfaeci_cysS-F | GCAGCCACCAATTTACAACGA | 86 |
|  |  | Ecfaeci_cysS-R | TCATCTGCCAAATTCTCTGAGG |  |
|  | *purD* | Ecfaeci_purD-F | GGAATGAAAGAACTAGGACGCT | 71 |
|  |  | Ecfaeci_purD-R | GGTCCTTCCTTCGTAGCAAT |  |
| ***P. acidilactici*** | *asnS* | Pdacidi_asnS-F | GCCATCCACGAATTCCTCCA | 80 |
|  |  | Pdacidi_asnS-R | GCACCTTCCGTATCACTAGAAGT |  |
|  | g1164 | Pdacidi_g1164-F | TTTAGGAGCAATCATCGCAATG | 75 |
|  |  | Pdacidi_g1164-R | CCAACTAATACCGCGCTACC |  |
| ***P. pentosaceus*** | *nagK* | Pdpento_nagK-F | GGGTGTCACTAACGGAGCAA | 107 |
|  |  | Pdpento_nagK-R | CAATTCCGCCCGCTTGTAAG |  |
|  | g4364 | Pdpento_g4364-F | TGCTTACCACCAACCTGCTT | 76 |
|  |  | Pdpento_g4364-R | TGGACGTTCTAGGTTTCCGT |  |
